# Impact of Lung Expansion Therapy Using Positive End-Expiratory Pressure in Mechanically Ventilated Patients Submitted to Coronary Artery Bypass Grafting

**DOI:** 10.21470/1678-9741-2019-0016

**Published:** 2019

**Authors:** André Luiz Lisboa Cordeiro, Sarah Carvalho, Maria Clara Leite, André Vila-Flor, Bruno Freitas, Lucas Sousa, Quetla Oliveira, André Raimundo Guimarães

**Affiliations:** 1 Escola Bahiana de Medicina e Saúde Pública (Bahiana) - Unidade Acadêmica Brotas, Salvador, Bahia, Brazil.; 2 Faculdade Nobre (FAN), Feira de Santana, Bahia, Brazil.; 3 Instituto Nobre de Cardiologia (INCARDIO), Feira de Santana, Bahia, Brazil.

**Keywords:** Coronary Artery Bypass, Hemodynamics, Airway Extubation, Lung, Positive-Pressure Respiration

## Abstract

**Objective:**

To evaluate the impact of different levels of positive end-expiratory pressure (PEEP) on gas exchange in patients undergoing coronary artery bypass grafting (CABG).

**Methods:**

A randomized clinical trial was conducted with patients undergoing CABG surgery. Patients were randomized into three groups: Group 10, PEEP of 10 cmH_2_O; Group 12, PEEP of 12 cmH_2_O; and Group 15, PEEP of 15 cmH_2_O. After the randomization, all patients underwent gas analysis at three moments: (1) before lung expansion therapy (LET); (2) 30 minutes after LET; and (3) one hour after extubation.

**Results:**

Sixty-six patients were studied, of which 61.7% were men, with mean age of 64 ± 8.9 years. Patients allocated to Group 15 showed a significant improvement in gas exchange comparing pre- and post-expansion values (239±21 *vs.* 301±19, P<0,001) and the increase was maintained after extubation (278±26). Despite the use of high levels of PEEP, no significant hemodynamic change was evidenced.

**Conclusion:**

It is concluded that high levels of PEEP (15 cmH_2_O) are beneficial for the improvement of gas exchange in patients undergoing CABG.

**Table:** 

Abbreviations, acronyms & symbols				
AMI	= Acute myocardial infarction		ICU	= Intensive care unit
ANOVA	= Analysis of variance		LET	= Lung expansion therapy
BMI	= Body mass index		MBP	= Mean arterial pressure
CABG	= Coronary artery bypass grafting		MV	= Mechanical ventilation
DBP	= Diastolic blood pressure		PEEP	= Positive end-expiratory pressure
DM	= Diabetes mellitus		PS	= Supportive pressure
EPAP	= Positive expiratory pressure		SaO_2_	= Arterial oxygen saturation
FiO_2_	= Inspired fraction of oxygen		SBP	= Systolic blood pressure
HR	= Heart rate		SPSS	= Statistical Package for the Social Sciences

## INTRODUCTION

Cardiovascular diseases, besides being highly prevalent, also present an exponential increase in their incidence due to modifiable and non-modifiable factors. The surgical treatment appears as a strategy to increase survival and improve quality of life, and coronary artery bypass grafting (CABG) is the most performed surgery^[1,2]^.

Despite this potential benefit, CABG is closely associated with a decline in ventilatory muscle strength and lung function. The reduction in lung function has a negative impact on the oxygenation of these patients, making them hypoxemic and increasing the risk of postoperative pulmonary complications^[3,4]^.

In this sense, the lung expansion therapy (LET) is an interesting alternative to reestablish the gas exchanges and, consequently, normalize the oxygenation^[5,6]^. The application of positive end-expiratory pressure (PEEP) is well established for this purpose, but the pressure required to correct hypoxemia is still questioned.

Borges et al.^[7]^ verified that the application of pressure with PEEP of 10 cmH_2_O was associated with improved gas exchange in patients submitted to CABG. In contrast, Lima et al.^[8]^ did not find a significant impact when they applied the same pressure as Borges before tracheal extubation. Therefore, in the literature on this subject, there is a difference regarding the values of PEEP used. The objective of this study was to evaluate the impact of different levels of PEEP on oxygenation in patients submitted to CABG.

## METHODS

This is a randomized clinical trial in patients admitted to the intensive care unit (ICU) of the Instituto Nobre de Cardiologia/Santa Casa de Misericórdia in Feira de Santana, Bahia, Brazil. Patients with ages from 30 to 80 years of both genders were submitted to CABG through median sternotomy and extracorporeal circulation. Patients with history of pneumopathy or emergency surgeries confirmed by medical report, who died during or after surgery, and those who did not agree to sign the Informed Consent Term were excluded. This study was approved by the Research Ethics Committee of Faculdade Nobre, with number 2.002.971.

Before surgical procedure, the patients’ clinical and surgical data were collected. Afterwards, the patients were referred to the surgical center and later to the ICU, where they were connected to the mechanical ventilator in volume-controlled mode, with a tidal volume of 6 ml/kg, respiratory rate of 15 incursions per minute, PEEP of 5 cmH_2_O, and inspired fraction of oxygen (FiO_2_) of 40%.

Patients began to interact with the ventilator and were placed in the supportive pressure (PS) mode with 7 cmH_2_O, PEEP of 5 cmH_2_O, and FiO_2_ of 40%. The mean time to start PS mode was 3±1 hours. When they met the criteria for extubation (ability to start the effort, hemodynamic and respiratory stability, bleeding controlled by the drains, absence of acid-base disorder, ability to interact with the environment through activities such as blinking or tongue out, and absence of edema in the airways), they were randomized by lottery to one of three groups: Group 10, where they remained in PS of 7 cmH_2_O and FiO_2_ of 40%, but PEEP was increased to 10 cmH_2_O; Group 12, with the same parameters of the previous group, but with an increase of PEEP to 12 cmH_2_O; and Group 15, which maintained the values of the other groups, but with PEEP of 15 cmH_2_O. These values were maintained for 30 minutes before extubation, and arterial samples for hemogasometry were collected at the end of this period. After LET, the patients were ventilated with the following parameters: PS of 7 cmH_2_O, PEEP of 5 cmH_2_O, and FiO_2_ of 40%, and extubation was made.

After and before LET, the patients’ vital signs, such as systolic blood pressure, diastolic blood pressure, mean heart rate, and heart rate monitor, were evaluated.

After extubation, the patients were continuously evaluated, and a low-flow oxygen carrier was used, with a release of 2 liters per minute for all groups. One hour after extubation, new hemogasometry was performed to evaluate the late impact of LET. Therefore, hemogasometry was performed before PEEP elevation, after 30 minutes of LET, and one hour after extubation.

The Statistical Package for the Social Sciences (SPSS) software, version 20.0, was used for statistical analysis. To evaluate the normality of the sample, the Shapiro-Wilk test was used. Categorical variables were analyzed using Chi-square test. LET and hemodynamics behavior were evaluated by analysis of variance (ANOVA), comparing the three groups. It was considered statistically significant if P<0.05.

## RESULTS

During the study period, 70 patients were submitted to cardiac surgery in the abovementioned hospital, of which 10 were excluded from this study due to the following: five due to the presence of pneumopathies, one due to emergency surgery, and four did not agree to sign the consent term. A total of 60 patients were included in the study, of which 37 (61.7%) were males, and the mean age was 64±8.9 years. No difference was observed between the groups, showing the homogeneity of the sample (Table 1).

**Table 1:** Clinical and surgical characteristics in different levels of PEEP of patients submitted to CABG.

Variable	PEEP 10 (n: 19)	PEEP 12 (n: 23)	PEEP 15 (n: 18)	*P*-value
Sex				0.56^a^
Male	11 (58%)	13 (57%)	11 (61%)	
Female	8 (42%)	10 (43%)	7 (39%)	
Age (years)	62±11	62±9	64±7	0.23^b^
BMI (kg/m^2^)	23±3	22±4	23±4	0.89^b^
Comorbidities				
Hypertension	12 (63%)	14 (61%)	12 (67%)	0.76^a^
DM	8 (42%)	7 (30%)	8 (44%)	0.60^a^
Dyslipidemia	7 (37%)	7 (30%)	8 (44%)	0.98^a^
Smoking	2 (11%)	3 (13%)	3 (17%)	0.78^a^
AMI	4 (21%)	3 (13%)	3 (17%)	0.87^a^
Extracorporeal circulation time (min)	63±17	63±18	67±20	0.45^b^
Bypass	2.5±0.7	2.8±0.5	2.3±0.5	0.22^b^
MV time (hours)	6±2	7 ± 3	6 ± 3	0.87^b^

a Chi-square test;

b analysis of variance (ANOVA); AMI=acute myocardial infarction; BMI=body mass index; CABG=coronary artery bypass grafting; DM=diabetes mellitus; MV=mechanical ventilation; PEEP=positive end-expiratory pressure

Table 2 shows the behavior of oxygenation index and arterial oxygen saturation (SaO_2_). The oxygenation index was similar between the groups before and after the PEEP increment; however, when the behavior of the pre and post-LET variables was analyzed, a significant increase in Group 15 was observed. On the other hand, SaO_2_ values did not change significantly between the pre and post-LET periods and in the comparison between the groups.

**Table 2:** Analysis of oxygenation in different levels of PEEP of patients submitted to CABG.

Variable	PEEP 10 (n: 19)	PEEP 12 (n: 23)	PEEP 15 (n: 18)	*P*^a^-value
Oxygenation index				
Before LET	230±23	246±17	239±21	0.93
After LET	250±19	262±19	301±19	0.03
After extubation	240±23	259±22	278±26	0.02
P^a^	0.45	0.65	<0.001	
SaO_2_ (%)				
Before LET	95±2.3	97±2	96±1.6	0.50
After LET	97±1.2	98±1.7	99±0.7	0.25
After extubation	96±1.9	97±1.4	98±1.1	0.54
*P*	0.66	0.38	0.49	

a analysis of variance (ANOVA); CABG=coronary artery bypass grafting; LET=lung expansion therapy; PEEP=positive end-expiratory pressure; SaO_2_=arterial oxygen saturation

When we verified the hemodynamic behavior during LET, a physiological pattern was observed without significant alterations between the analyzed groups (Table 3).

**Table 3:** Hemodynamic impact of different levels of PEEP in patients submitted to CABG.

Variable		PEEP 10 (n: 19)	PEEP 12 (n: 23)	PEEP 15 (n: 18)	*P*^a^-value
SBP (mmHg)	Before LET	114±15	121±16	125±17	0.08
	After LET	106±8	111±17	119±17	0.28
	*P*^b^	0.06	0.16	0.35	
DBP (mmHg)	Before LET	60±7	64±3	65±6	0.50
	After LET	57±7	61±9	59±7	0.28
	*P*^b^	0.41	0.35	0.48	
MBP (mmHg)	Before LET	78±11	83±9	85±9	0.59
	After LET	73±8	78±11	79±9	0.76
	*P*	0.78	0.88	29	
HR (bpm)	Before LET	104±9	102±21	104±8	0.86
	After LET	103±7	99±12	100±12	0.76
	*P*	0.31	0.56	0.24	

a analysis of variance (ANOVA);

b paired Student's t-test; CABG=coronary artery bypass grafting; DBP=diastolic blood pressure; HR=heart rate; LET=lung expansion therapy; MBP=mean arterial pressure; PEEP=positive end-expiratory pressure; SBP=systolic blood pressure

## DISCUSSION

Based on the findings, we found out that the use of PEEP of 15 cmH_2_O for LET was associated with a significant improvement in the oxygenation rate that remained even after one hour of extubation in patients undergoing CABG. Despite the use of higher pressures than that, the hemodynamic impact was not significant and was not associated with the need to increase vasoactive drugs during therapy.

In a study published by Borges et al.^[7]^, different levels of PEEP (5, 8, and 10 cmH_2_O) were used in patients undergoing myocardial revascularization surgery, and a better oxygenation index was evidenced in the group of pressurized patients with PEEP of 10 cmH_2_O. In the present study, there was also an increase in oxygenation with PEEP of 10 cmH_2_O, but with values lower than 15 cmH_2_O. In the study by Borges et al.^[7]^, it was also verified an optimization of compliance, which was not evaluated in this study.

Despite these findings, Lima et al.^[8]^ did not verify the effects of the use of different levels of PEEP on the oxygenation of patients submitted to CABG. It is noteworthy that in the Lima study, PEEP reached a maximum value of 10 cmH_2_O, while in the present study it reached a value of 15 cmH_2_O, which was associated with an improvement of this outcome.

The probable explanation for this divergence of results lies in the methodological differences of the studies and the level of PEEP applied in these patients. The application of high PEEP (15 cmH_2_O) is associated with an improvement in the aeration of the dependent lung areas and the promotion of more effective collateral ventilation. Thus, there is an optimization of the ventilation/perfusion ratio and, consequently, an improvement in oxygenation^[9]^.

In their review, Padovani et al.^[10]^ showed that for the treatment of hypoventilation and post-surgical hypoxemia most of the studies used alveolar recruitment maneuvers with PEEP values reaching 45 cmH_2_O. A limitation for this type of technique is the possibility of hypotension during the maneuver, and LET is as effective as these maneuvers, but without the hemodynamic repercussions, as demonstrated in this study.

In another study using recruitment, an improvement in oxygenation and a decrease in the incidence of atelectasis were observed in patients who received a PEEP of 20 cmH_2_O^[11]^.

Dongelmans et al.^[12]^ conducted a retrospective analysis of two clinical trials dividing patients into two groups: the first started with a PEEP of 5 cmH_2_O that was maintained throughout the weaning process; and the other received a PEEP of 10 cmH_2_O in the first four hours and of 5 cmH_2_O until the time of extubation. There was an improvement in gas exchange in the group that received an increase in positive pressure, but this group also stayed longer in mechanical ventilation.

Another relevant point of the present study is the maintenance of oxygenation even after one hour of extubation. The group of patients who used PEEP of 15 cmH_2_O maintained the oxygenation index above the others with the same level of post-extubation oxygen therapy. Although not evaluated, this maintenance of the exchanges may be related to the reduction of the need for oxygen use and a reduction in postoperative complications, since hypoxemia becomes a risk factor for these complications.

The peripheral oxygen saturation in the present study did not change during LET, differently from the behavior of this variable in the study by Pettersson et al.^[13]^ who demonstrated an increase in saturation and other variables after reexpansive techniques.

In a recent study, Cordeiro et al. demonstrated that this application of high levels of PEEP also improved oxygenation during the application of therapy in non-invasive ventilation in patients after CABG^[14]^.

A concern regarding LET in this patient profile is hemodynamic instability with the need to increase the flow rate of vasoactive drugs. Méndez et al.^[15]^ did not verify hemodynamic changes when they compared patients after heart surgery receiving PEEP of 5 or 12 cmH_2_O. This corroborates with the results of the present study and ratifies the safety of the pre-extubation technique.

Similar results were also found by Sena et al.^[16]^, but safety was demonstrated with patients already extubated and receiving the application of positive expiratory pressure (EPAP) of 10 cmH_2_O, generating a discrete impact on heart rate.

In the present study, there was a tendency to reduce hemodynamic variables during LET, but some points should be observed: independently of the pressure level, there was a mild hemodynamic impact; despite this alteration, no adverse events occurred and no patient needed interruption of therapy; there was no need for increment of vasoactive amines to divide the use of positive pressure. We thus demonstrated that the therapy, besides beneficial to increase the oxygenation variable, is also safe and has no associated adverse events.

### Limitation

As limitations of the present study it is possible to emphasize the lack of a sample calculation, the non-application of a severity scale of the operated patients, and the absence of an outcome, such as post-extubation complications.

## CONCLUSION

Based on the findings, we found out that the use of PEEP of 15 cmH_2_O for LET was associated with a significant improvement in the oxygenation rate that remained even after one hour of extubation in patients undergoing CABG.

**Table:** 

Author's roles & responsibilities	
ALLC	Substantial contributions to the conception or design of the work; or the acquisition, analysis, or interpretation of data for the work; drafting the work or revising it critically for important intellectual content; agreement to be accountable for all aspects of the work in ensuring that questions related to the accuracy or integrity of any part of the work are appropriately investigated and resolved; final approval of the version to be published
SC	Substantial contributions to the conception or design of the work; or the acquisition, analysis, or interpretation of data for the work; final approval of the version to be published
MCL	Substantial contributions to the conception or design of the work; or the acquisition, analysis, or interpretation of data for the work; final approval of the version to be published
AVF	Substantial contributions to the conception or design of the work; or the acquisition, analysis, or interpretation of data for the work; final approval of the version to be published
BF	Substantial contributions to the conception or design of the work; or the acquisition, analysis, or interpretation of data for the work; final approval of the version to be published
LS	Substantial contributions to the conception or design of the work; or the acquisition, analysis, or interpretation of data for the work; final approval of the version to be published
QO	Substantial contributions to the conception or design of the work; or the acquisition, analysis, or interpretation of data for the work; final approval of the version to be published
ARG	Substantial contributions to the conception or design of the work; or the acquisition, analysis, or interpretation of data for the work; final approval of the version to be published
